# A retrospective evaluation of patients with sleep breathing disorders in single center, Edirne province

**DOI:** 10.5578/tt.20239704

**Published:** 2023-09-22

**Authors:** Y. İlker, C. KORUCUOĞLU, E. YILDIZ, G. ALTIAY

**Affiliations:** 1 Clinic of Pulmonology, Babaeski State Hospital, Kırklareli, Türkiye; 2 Department of Pulmonology, Trakya University Faculty of Medicine, Edirne, Türkiye

**Keywords:** sleep apnea, comorbidity, obesity, device compliance

## Abstract

**ABSTRACT:**

A retrospective evaluation of patients with sleep breathing disorders in single center, Edirne province

**Introduction:**

Our aim was to investigate the characteristics of patients with preliminary diagnosis of sleep breathing disorder studied in Edirne province, to identify the risk factors and comorbid conditions, to determine the diagnostic distribution of our sleep patients by analyzing the results of polysomnography and PAP titration, and to understand their problems related to treatment compliance and device use.

**Materials and Methods:**

Our study retrospectively evaluated the results of 956 patients who had sleep-related complaints in our region and underwent full nocturnal polysomnography and PAP titration with a preliminary diagnosis of sleep breathing disorder.

**Results:**

A total of 956 patients were enrolled in the study, of whom 641 (67.1%) were males and 315 (32.9%) were females. Of our patients, 49.7% had severe obstructive sleep apnea (OSA), 18.2% had moderate OSA, 17.9% had mild OSA, 11.4% had REM-dependent OSA, and 8.4% had positionsupine-dependent OSA. Hypertension was the most common comorbid condition in 44% of our patients, and diabetes mellitus in 25%. It was determined that 228 (57.9%) of 394 patients who were recommended to use the PAP device received the device, and 71.5% of these patients could use the device in a compatible manner.

**Conclusion:**

Patients with appropriate symptoms can be diagnosed with a high probability of OSA in our region. The fact that a substantial group of patients diagnosed with OSA and recommended to use the PAP device did not receive the device or the proportion of those who could not use the device was high is one of the notable findings of the study

## Abstract (tr)

**ÖZ:**

Edirne ilinde tek merkezde uykuda solunum bozukluğu olan hastaların retrospektif olarak değerlendirilmesi

**Giriş:**

Edirne ilinde uykuda solunum bozukluğu ön tanısı ile tetkik edilen hastaların özelliklerini inceleyip, risk faktörü ve eşlik eden hastalıkları belirlemek, polisomnografi ve PAP titrasyonu sonuçlarımızı irdeleyerek hastalarımızın tanısal dağılımını, tedavi uyumu ve cihaz kullanımı ilişkili sorunlarını anlamak amacı ile hastalarımızın değerlendirmesi yapılmıştır.

**Materyal ve Metod:**

Çalışmamızda bölgemizde uyku ilişkili yakınmaları mevcut olup uykuda solunum bozukluğu ön tanısıyla tüm gece polisomnografi ve PAP titrasyonu yapılan 956 hastanın sonuçları retrospektif olarak değerlendirilmiştir.

**Bulgular:**

Çalışmaya toplam 956 hasta alınmış olup, bu hastalardan 641 (%67,1)’i erkek, 315 (%32,9)’i kadın idi. Hastalarımızın %49,7’si ağır OSA, %18,2’si orta OSA, %17,9’u hafif OSA, %11,4’ü REM bağımlı OSA, %8,4’ü pozisyon-supin bağımlı OSA olarak kabul edildi. Hastalarımızın %44’ünde hipertansiyon ve %25’inde diabetes mellitus en sık saptanan komorbid durumlar idi. PAP cihazı kullanımı önerilen 394 hastanın 228 (%57,9)’inin cihazı aldığı ve bu hastaların %71,5’nin cihazı uyumlu olarak kullanabildi- ği saptandı.

**Sonuç:**

Bölgemizde ilişkili semptomlar ile başvuran hastalara yüksek oranda OSA tanısı konulabilmektedir. OSA tanısı konmuş ve PAP cihazı önerilen önemli bir hasta grubunun cihazı almamış olması veya alıp da cihaza uyum göstermeyenlerin oranının fazla oluşu çalışmanın dikkat çekici sonuçlarındandır.

## INTRODUCTION

Sleep is a reversible, transient, partial, periodic loss of the organism’s communication with the environment due to stimuli of varying intensity. Diseases and deaths caused by respiratory disorders during sleep are referred to as sleep breathing disorders (SBD).

According to the reorganized classification of sleep disorders (ICSD-3) of 2014 American Academy of Sleep Medicine (AASM), obstructive sleep apnea (OSA) is the most common form in the spectrum of sleep breathing disorders ( 1 ). According to the International Classification of Sleep Disorders, OSA is defined as a syndrome characterized by recurrent episodes of complete (apnea) or partial (hypopnea) upper airway obstruction during sleep and often by a decrease in blood oxygen saturation ( 1 , 2 ). Obstructive sleep apnea is an important public health problem with high morbidity and mortality, characterized by complete or partial cessation of breath during sleep, excessive daytime sleepiness, and snoring, and its consequences affect all systems, especially the cardiovascular system. Compared with previous years, the incidence of this condition is increasing due to the growing awareness and widespread use of sleep laboratories ( 3 ).

Treatment goals for sleep apnea are to relieve symptoms of the disease, improve sleep quality, and normalize AHI (hourly apnea and hypopnea counts during sleep) and nocturnal oxygen saturation. Potential outcomes of successful sleep apnea treatment include clinical improvements (reduced daytime sleepiness, fresher morning awakenings, etc.), reduced need for health care services, reduced cardiovascular disease, and reduced mortality. Sleep apnea should be treated with a long-term and multidisciplinary management approach.

In this study, we retrospectively analyzed the records of 956 patients who presented to the Chest Diseases Outpatient Clinic at Trakya University Faculty of Medicine with symptoms of sleep disorders and underwent polysomnography (PSG). Our aim in the study was to investigate the characteristics of sleep patients in our region, identify the risk factors and comorbid conditions, determine the diagnostic distribution of our sleep patients by examining our polysomnography and CPAP-BPAP-titration results, and understand their problems related to treatment compliance and device use.

## MATERIALS and METHODS

Our study retrospectively analyzed the records of all patients who presented to the Chest Diseases Sleep outpatient clinic of Trakya University Medical Faculty Hospital with sleep symptoms and underwent nocturnal polysomnography (Comet-Grass Technologies Astro-Med, West Warwich, Rhode Island, USA and Compumedics System) or CPAP and BPAP titration with a preliminary diagnosis of sleep breathing disorder within a time period of eight years. Patients who could not undergo full nocturnal polysomnography and patients who made an appointment but did not appear for polysomnography were excluded from our study.

Sleep-related symptoms such as snoring, witnessed apnea, excessive daytime sleepiness, and Epworth Sleepiness Scale scores were recorded from patients’ files. Patient demographic characteristics such as age, sex, height, weight, body mass index, and neck circumference were also recorded. Apnea-hypopnea index, lowest oxygen saturation value, mean desaturation, and comorbid conditions were noted based on the results of patients who had undergone nocturnal polysomnography. Patients’ diagnoses resulting from PSG and the devices given to patients were retrospectively reviewed. The status of device use of patients for whom a device report had been issued was questioned.

Our study was conducted with the approval of the Ethics Committee of the Trakya University Faculty of Medicine with the decision dated 27/04/2016 and numbered 08/16.

### Evaluation of PSG Findings

Apnea-hypopnea index (AHI), lowest oxygen saturation value and mean desaturation percentages, diagnosis, and treatment recommendations in the results section were recorded in the patients’ polysomnography reports. Polysomnography findings with 30-second epochs were evaluated according to the guideline recommendations of the AASM. In the presence of accompanying symptoms and comorbid conditions, AHI was classified as mild OSA if the score was 5-15, moderate OSA if it was 15-30, and severe OSA if it was above 30. In our patients diagnosed with OSA (total AHI≥ 5), a value of REM-AHI, which was at least twice or higher than the value for NonREM-AHI, was considered REM-related OSA, provided that the value for NonREM- AHI was within normal limits (<5). The same relationship with position was considered position-dependent OSA.

### Statistical Methods

SPSS v.22 package program was used for data analysis. Descriptive statistics were shown as mean ± standard deviation (SD) or median (minimum-maximum) for continuous variables and frequency and (%) for categorical variables. The Kolmogorov-Smirnov and Shapiro-Wilk tests were used to determine whether the distribution of variables conformed to the normal distribution. As a result of the analyses for “demographic data (sex, age, etc.)”, the data were given as frequency and (%) for categorical variables and as mean ± SD (SD) or median (minimum-maximum) for continuous variables.

In the correlation analysis between body mass index (BMI) and continuous variables of AHI, Spearman correlation analysis was chosen because of nonconformity with normal distribution, and the results were given as tables and comments.

Diagnostic decision characteristics of the AHI value in relation to the cut-off value of 10 of the EPWORTH value were examined using ROC (Receiver Operating Characteristics) analysis. In the presence of significant cut-off values, the sensitivity, specificity, positive predictive value, and negative predictive value of these cut-off values were calculated. When evaluating the area under the curve, values below p< 0.05 were considered and interpreted as statistically significant

## RESULTS

A total of 956 patients were enrolled in the study, of whom 641 (67.1%) were males and 315 (32.9%) were females. Mean age of the patients participating in the study at the time of diagnosis was 51.31 years (SD ± 11.37); median age was 52 years (min 18-max 88).

The age groups of patients participating in the study at the time of diagnosis were as follows: 149 subjects (15.6%) were in the age group <40 years old, 571 subjects (59.7%) were in the age group between 40-59.9 years old, 233 subjects (24.4%) were in the age group between 60-79.9 years old, and three subjects (0.3%) were in the age group ≥80 years old.

When mean BMI values of the study participants were evaluated, the overall mean BMI was 33.75 (SD ± 6.73). Median BMI value was 32.45, minimum BMI value was 16.53, and maximum BMI value was 67.60. According to the classification of obesity obtained by grouping BMI, 58 subjects (6.1%) belonged to the normal weight group (BMI≤ 25), 256 subjects (26.8%) belonged to the overweight group (BMI= 25-30), 486 subjects (50%) belonged to the obese group (BMI= 30-40), and 148 subjects (15.5%) belonged to the morbidly obese group (BMI≥ 40) ( Table 1 ).

Mean AHI of our patients was 38.24 (SD ± 31.21), and median AHI was 30.80 (min= 0-max= 72). In the diagnostic distribution of patients according to AHI values, 475 subjects (49.7%) were evaluated as severe OSA, 174 (18.2%) as moderate OSA, 171 (17.9%) as mild OSA, 109 (11.4%) as REM-dependent OSA, and 80 subjects (8.4%) as position-supine-dependent OSA ( Table 2 ).

Hypertension (HT) with a rate of 44% and diabetes mellitus (DM) with a rate of 25% were the most common comorbid conditions in our patients. Other comorbid conditions were coronary artery disease (CAD), chronic obstructive pulmonary disease (COPD), hyperlipidemia (HPL), congestive heart failure (CHF), depression, hypothyroidism, and asthma ( Table 3 ).

BMI distribution was calculated by age group. Mean BMI in the age group <40 years old was 30.68 (SD ± 6.37), mean BMI in the age group between 40-59.9 years old was 33.84 (SD ± 6.69), mean BMI in the age group between 60-79.9 years old was 33.98 (SD ± 6.65), and mean BMI in the age group ≥80 years old was 28.79 (SD ± 7.31) ( Table 4 ).

BMI distribution was calculated according to age groups. Mean BMI in the age group <40 years old was 30.68 (SD ± 6.37), mean BMI in the age group between 40-59.9 years old was 33.84 (SD ± 6.69), mean BMI in the age group between 60-79.9 years old was 33.98 (SD ± 6.65), and mean BMI in the age group ≥80 years old was 28.79 (SD ± 7.31) ( Table 4 ).

Between BMI and AHI values;

In the correlation analysis performed on the basis of the whole group, a statistically significant (p< 0.001) moderate correlation (R= 0.401) was found.

• In the correlation analysis performed in the severe OSA group, a statistically significant (p< 0.001) moderate correlation (R= 0.339) was found.

• In the correlation analysis performed in the morbidly obese group, a statistically significant (p= 0.006) low correlation (R= 0.226) was found.

The distribution of 474 patients in the severe OSA group was examined in terms of complaints of snoring, daytime sleepiness, and witnessed apnea, and up to 75% of the patients had three symptoms ( Table 5 ).

Diabetes mellitus, hypertension, and coronary artery disease were common comorbid conditions in patients diagnosed with OSA. While HT was the most common comorbid condition in 27.2% of our patients, the coexistence of HT and DM was found in 12.6%. In 44.5% of the patients, no HT, DM, or CAD was detected ( Table 6 ).

When evaluating the use of PAP devices in patients with OSA, it was determined that 228 (57.9%) of 394 patients who were recommended to use PAP devices received them. One hundred and sixty-three (71.5%) of the patients who received the device used it at least five days per week. Among these patients, the mean duration of device use was 6.5 hours/night (SD ± 1.60).

According to the analysis results of all patients, the lowest mean O2 saturation was 77.52, while it was 76.95 in the group with OSA. While the mean desaturation value in the overall group was 6.44, it was 6.70 in the group with OSA.

**Figure 1:**
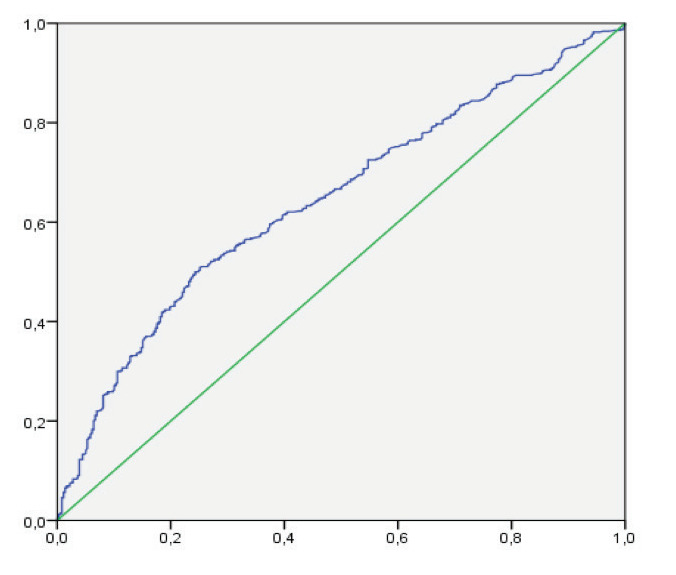
AHI Score- EPWORTH (10) ROC Analysis

After dividing the EPWORTH value into two groups above and below 10, a ROC analysis was performed to answer the question of whether a cut-off point for the AHI score could be established ( Figure 1 ). The area under the curve as the result of ROC-analysis was evaluated in terms of sensitivity, specificity, positive predictive value, and negative predictive value, and the results are shown in Table 7 .

According to the ROC analysis results, the highest values for sensitivity and specificity were obtained at a cut-off value of 42.8 for the AHI score, and sensitivity (50%) and specificity (75.7%) were determined. Furthermore, positive predictive value was 76.4% and negative predictive value was 49.0% ( Table 7 ).

**Table 1:** General characteristics of the patients

		n	%
Sex	Male	641	671
	Female	315	32.9
Age	Mean ± SD	51.31 ± 11.37	
	Med. (min-max)	52 (8-88)	
Age groups	<40 years old	149	15.6
	Between 40-59.9 years old	571	59.7
	Between 60-79.9 years old	233	24.4
	≥80 years old	3	0.3
BMI	Mean ± SD	33.75 ± 6.73	
	Med. (min-max)	32.45 (16.53-67.60)	
Weight distribution	Normal (<25)	58	6.1
	Overweight (25-30)	256	26.8
	Obese (30-40)	486	50.8
	Morbidly Obese (>40)	148	15.5
Neck circumference (Man)	Mean ± SD	43.57 ± 3.91	
	Med. (min-max)	43 (30-57)	
Neck circumference (Woman)	Mean ± SD	38.80 ± 3.96	
	Med. (min-max)	38.75 (29-53)	
Neck circumference (Male)	Neck circumference ≥42	430	67.1
	Neck circumference <42	211	32.9
Neck circumference (Female)	Neck circumference ≥38	182	57.8
	Neck circumference <38	133	42.2

BMI: Body mass index.

**Table 2:** Diagnoses of the patients

Diagnosis	n	(%)
Severe OSA	475	49.7
Moderate OSA	174	18.2
Mild OSA	171	17.9
REM-dependent OSA	109	11.4
Supine-dependent OSA	80	8.4
Normal sleep	54	5.6
Simple snoring	50	5.2
Supine ve REM-dependent OSA	48	5
Obesity hypoventilation syndrome	46	4.8
Overlap syndrome	12	1.3
OSA + Central apnea	6	0.6
UARS	4	0.4
Kyphoscoliosis	3	0.3
Could not diagnose	3	0.3

OSA: Obstructive sleep apnea, UARS: Upper airway resistance syndrome.

**Table 3:** Comorbid conditions accompanying patients

	Condition	n	%
HT	ABSENT	535	56.0
	PRESENT	421	44.0
DM	ABSENT	760	79.5
	PRESENT	196	20.5
Asthma	ABSENT	861	90.1
	PRESENT	95	9.9
CAD	ABSENT	866	90.6
	PRESENT	89	9.3
COPD	ABSENT	871	91.1
	PRESENT	85	8.9
HPL	ABSENT	883	92.4
	PRESENT	73	7.6
CHF	ABSENT	871	91.1
	PRESENT	84	8.8
Depression	ABSENT	907	94.9
	PRESENT	49	5.1
Hypothyroidism	ABSENT	911	95.3
	PRESENT	45	4.7
A history of nose surgery	ABSENT	890	93.1
	PRESENT	66	6.9

HT: Hypertension, DM: Diabetes mellitus, CAD: Coronary artery disease, COPD: Chronic obstructive pulmonary disease, HPL: Hyperlipidemia, CHF: Congestive heart failure.

**Table 4:** Age groups-BMI distribution

	Condition	n	%
Age groups	<40 years old	149	15.6
	Between 40-59.9 years old	571	59.7
	Between 60-79.9 years old	233	24.4
	≥80 years old	3	0.3
<40 years old	Mean ± SD	30.68 ± 6.37	
	Med. (min-max)	29.71 (16.53-51.26)	
<40 years old	Normal (<25)	21	14.1
	Overweight (25-30)	56	37.6
Obese (30-40)	55	36.9
Morbidly obese (>40)	14	39.4
Total	146	98.0
Between 40-59.9 years old	Mean ± SD	33.84 ± 6.69	
	Med. (min-max)	32.85 (18.65-67.60)	
Between 40-59.9 years old	Normal (<25)	23	4.0
	Overweight (25-30)	148	25.9
Obese (30-40)	303	53.1
Morbidly obese (>40)	94	16.5
Total	568	99.5
Between 60-79.9 years old	Mean ± SD	33.98 ± 6.65	
	Med. (min-max)	33.05 (18.73-60.09)	
Between 60-79.9 years old	Normal (<25)	13	5.6
	Overweight (25-30)	51	21.9
Obese (30-40)	127	54.5
Morbidly obese (>40)	40	17.2
Total	231	99.1
≥80 years old	Mean ± SD	28.79 ± 7.31	
	Med. (min-max)	29.38 (21.19-35.80)	
≥80 years old	Normal (<25)	1	33.3
	Overweight (25-30)	1	33.3
Obese (30-40)	1	33.3
Morbidly obese (>40)	0	0
Total	3	100

BMI: Body-mass index.

**Table 5:** Patients’ complaints

Complaints	n	%
Snoring, daytime sleepiness, witnessed apnea	357	75.2
Snoring and witnessed apnea	58	12.2
Snoring and daytime sleepiness	35	7.5
Snoring only	17	3.6
Daytime sleepiness and witnessed apnea	3	0.7
Daytime sleepiness only	2	0.4
There is none	2	0.4
Total	474	100

**Table 6:** Comorbid conditions in patients diagnosed with OSA

	Condition	n	%
DM-HT-CAD	HT only	147	27.2
	HT + DM	68	12.6
	DM only	31	5.7
	HT + CAD	20	3.7
	HT + DM + CAD	20	3.7
	CAD only	11	2.0
	DM + CAD	3	0.6
	There is none	241	44.5
	Total	541	100.0
HT-COPD	ABSENT	508	93.9
	PRESENT	33	6.1
	Total	541	100.0
CHF-COPD	ABSENT	529	97.8
	PRESENT	12	2.2
	Total	541	100.0
HT-CHF	ABSENT	498	92.1
	PRESENT	43	7.9
	Total	541	100.0

OSA: Obstructive sleep apnea, HT: Hypertension, DM: Diabetes mellitus, CAD: Coronary artery disease, COPD: Chronic obstructive pulmonary disease, CHF: Congestive heart failure.

**Table 7:** The relation between Epworth score and AHI

		EPWORTH Value		
AHI CUT-OFF 42.8		EPWORTH> 10 (Significant)	EPWORTH≤ 10 (Not significant)	Total
AHI≥ 42.8	Number of subjects	282	87	369
	Row percentage	76.4%*	23.6%	100.0%
	Column percentage	50.0%**	24.3%	40.0%
AHI< 42.8	Number of subjects	282	271	553
	Row percentage	51.0%	49.0%***	100.0%
	Column percentage	50.0%	75.7%****	60.0%
Total	Number of subjects	564	358	922
	Row percentage	61.2%	38.8%	100.0%
	Column percentage	100.0%	100.0%	100.0%

*Positive predictive value, **Sensitivity, ***Negative predictive value, ****Specifity. AHI: Apnea-hypopnea index.

## DISCUSSION

The main conclusion drawn from our study is that the very high rate of diagnosis of OSA in the patients who presented to our clinic with sleep-related complaints and underwent polysomnography shows the importance of high clinical suspicion in the outpatient clinics. Hypertension and obesity were the most common comorbidities in our patients. A significant relation was found between AHI and BMI. The most important conclusion of our study that, in our opinion, should be emphasized and needs a solution is that the rate of patients obtaining and using PAP devices effectively is low and therefore, we cannot treat those patients.

Obstructive sleep apnea syndrome is the most common sleep-related breathing disorder. Although OSA is most common in older men, it also affects women and children. Because of the different definitions of OSA, different prevalence rates can be reported. When OSA is generally defined as apnea-hypopnea index (AHI) of more than five events per hour of sleep, the prevalence in the United States is approximately 15-30% in men and 10-15% in women. In the presence of AHI≥ 5 events and accompanying symptoms or AHI≥ 15 per hour, the estimated prevalence is about 15% in men and 5% in women ( 4 , 5 , 6 , 7 ). Obstructive sleep apnea syndrome is about two to three times more common in men than in women, with a similar risk in women with increasing age ( 7 , 8 ). In the review study by Karl A. Franklin et al., which evaluated 11 studies between 1993 and 2013, the prevalence of OSA was 22% in men and 17% in women, when AHI was accepted as ≥5. When AHI≥ 5 and symptoms such as excessive daytime sleepiness were considered, this rate was 6% in men and 4% in women ( 9 ).

In our study, mean age was 51.3 years (SD ± 11.37). Many studies investigating the relation between age and OSA risk have shown that OSA prevalence increases until the 6th-7th decade of life and then reaches a plateau ( 4 , 7 , 10 ). In our study, 571 subjects (59.7%) were in the 40- to 59-year-old age group and 233 subjects (24.4%) were in the 60- to 79-year-old age group, and the prevalence of OSA ranged from 40 to 60 years.

When patients were classified according to the severity and type of OSA, 475 subjects (49.7%) belonged to the severe OSA group, 174 subjects (18.2%) belonged to the moderate OSA group, 171 subjects (17.9%) belonged to the mild OSA group, 109 subjects (11.4%) belonged to the REM-dependent OSA group, 80 subjects (8.4%) belonged to the supine-dependent OSA group, and 48 subjects (5%) belonged to the supine- and REM-dependent OSA group. In other studies from Türkiye, a higher rate of patients with severe OSA has been diagnosed ( 11 , 12 ). The fact that in our study, the number of patients with severe OSA was higher than those with mild and moderate OSA suggests that patients seek medical attention only when the severity of their symptoms increases and at later stages.

Obesity is one of the most important risk factors for OSA, and this relation has been clearly shown in many studies ( 4 , 13 , 14 ). In our study, mean body mass index (general BMI mean) was 33.75 (SD ± 6.73), 58 subjects (6.1%) in the normal-weight group had a BMI< 25, 256 subjects (26,8%) in the overweight group had a BMI= 25-30, 486 subjects (50.8%) in the obese group had a BMI= 30-40, and 148 subjects (15.5%) in the morbidly obese group had a BMI> 40.

There is a stronger relation between increased neck thickness and OSA than obesity. Neck thickness greater than 17 inches (43 cm) in males and 16 inches (40 cm) in females is related to significantly increased OSA ( 15 ). Hoffstein et al. and Katz et al. ( 16 , 17 ) have also found that BMI and neck circumference were significantly higher in apnea patients than in nonapnea patients. In our study, mean neck circumference was 43.57 ± 3.91 in males and 38.80 ± 3.96 in females. Neck circumference was ≥42 cm in 430 patients (67.1%) in males and ≥38 cm in 182 patients (57.8%) in females.

In patients with OSA, obesity is often accompanied by cardiovascular and metabolic disorders. In our study, a high obesity rate of 66.3% was observed. Hypertension, DM, asthma, CAD, COPD, CHF, hyperlipidemia, and depression were found in 44%, 20.5%, 9.9%, 9.3%, 8.9%, 8.8%, 7.6%, and 5.1%, respectively. In addition, 6.9% of our patients had a history of nose surgery. In a study by Uyar et al, HT was found in 35.5% of cases, DM in 11.3%, and CAD in 16% ( 18 ). Obstructive sleep apnea syndrome causes long-term cardiovascular complications, of which hypertension is the most common. In our study, hypertension was the most common comorbid condition.

In the study by Duran et al., blood pressure values have also been found significantly higher in patients with suspected OSA ( 19 ). In our study, BMI was shown to increase with age. Our study showed that mean BMI was 30.68 ± 6.37 in patients younger than 40 years old, 33.84 ± 6.69 between 40 and 60 years old, and 33.98 ± 6.65 between 60 and 80 years old. Body mass index increases with age, and a positive correlation was shown between BMI and AHI. In our study, the severity of OSA was found to increase with obesity

Major symptoms of OSA are snoring, witnessed apnea, and excessive daytime sleepiness. In our study, snoring was the most common complaint (98.6%), especially in severe OSA, and the proportion of patients with three major symptoms was 72.5%.

The gold standard treatment method for obstructive sleep apnea syndrome is PAP. PAP therapy is recommended for all patients diagnosed with OSA. There is sufficient evidence that PAP therapy reduces sleep-related breathing events, daytime sleepiness, and accident risk, improves systemic blood pressure, and increases quality of life in patients with OSA with various conditions.

In 394 of our patients diagnosed with OSA, PAP treatment (CPAP-BPAP) was recommended. Assessment of 283 of these patients during their outpatient visits and by telephone revealed that 228 patients (80.5%) received the device and 163 (71.5%) used it.

The inability of patients to comply with treatment diminishes the potential benefit of PAP therapy. In OSA patients, the use of the PAP device for less than four hours on at least 70% of nights (five days per week) is defined as noncompliance. Studies of PAP compliance have found compliance rates ranging from 28% to 83% due to some definitional differences. Compliance rates are 65-90% if only patient utterances are considered. When the memory card counting system is used on the PAP device, the compliance rate drops to 46% in patients using PAP ( 20 ).

Many studies have shown that using PAP for at least four hours per night reduces daytime sleepiness and is related to improved quality of life, neurocognitive function, cardiovascular disease, and diabetes; hence the four-hour threshold is used ( 21 , 22 , 23 ). Some researchers state that PAP should be used at least six hours per night, six nights per week, to ensure adequate oxygen saturation and sleep integrity during sleep for adequate adaptation and to eliminate daytime symptoms. However, it is generally accepted that the more frequently it is used, the greater the effect ( 24 , 25 ).

Identifying the risk factors that affect treatment compliance and intervening appropriately to ensure treatment compliance is critical. Factors affecting treatment compliance may be due to the patient, the device mask, or other factors. Informing the patient in detail about the severity of the disease and the results will ensure that the patient receives the device and uses it regularly. Close follow-up of treated patients at the end of the first month and as needed also increases long-term compliance ( 26 ).

In our study, most patients did not receive the device because of financial reasons, and some thought they could not use it. There are also a considerable number of patients who do not submit their reports and do not come for check-ups. On the other hand, patients who cannot use the device often feel disturbed by the mask, have high blood pressure, dry mouth and irritated nose, develop choking attacks and claustrophobia, snore-witnessed apnea symptoms do not regress despite using the device, feel uncomfortable in hot weather, sweat excessively, change positions in bed frequently at night or have difficulty using the device during frequent urination, and their wives feel disturbed by the loud operation of the device. Some patients stopped using the device due to weight loss or regression of their symptoms after nose surgery.

Mean duration of device use in our patients was 6.50 hours ± 1.60 hours. CPAP usage duration was consistent with the literature data. Symptomatic patients benefited from the device to a greater extent, and most of our patients who received the device used it regularly (at least five days per week). However, the biggest problem for patients was that they did not receive adequate information and education after receiving the device and did not know exactly how to deal with the problems that arose. In addition, most of our patients did not visit the outpatient clinic regularly after receiving the device. The presence of patients who discontinued the use of the device due to device malfunction, even though they used the device regularly, was an indication of the importance of patient communication and education.

## CONCLUSION

Patients with symptoms of sleep breathing disorder may be diagnosed with a high rate of OSA. It is critical that patients be screened for OSA, along with accompanying symptoms and risk factors, especially in the presence of comorbid conditions. The study’s notable findings are that a significant group of patients diagnosed with OSA who were recommended a PAP device did not receive the device, or the rate of those who did not use the device was high. Until the problems associated with the provision and use of the PAP device, which is the gold standard in the treatment of OSA, are resolved, all efforts to diagnose the disease will be in vain.

## Ethical Committee Approval

This study was approved by the Trakya University Scientific Research Ethics Committee (Decision no: 08/16, Date: 27.04.2016).

## CONFLICT of INTEREST

The authors declare that they have no conflict of interest.

## AUTHORSHIP CONTRIBUTIONS

Concept/Design: All of authors

Analysis/Interpretation: All of authors

Data acqusition: CK, GA

Writing: All of authors

Clinical Revision: All of authors

Final Approval: All of authors

## References

[bb0005] American Academy of Sleep Medicine (2014). International classification of sleep disorders, 3rd ed. Darien, IL: AASM.

[bb0010] Köktürk O. (2008). Classification of sleep breathing disorders,
definitions and obstructive sleep apnea syndrome
(Epidemiology and clinical findings). Türkiye Klinikleri J Pulm Med-Special Topics.

[bb0015] Köktürk O. (2000). Obstructive sleep apnea syndrome results. Tuberc Thorac J.

[bb0020] Kline L.R. Clinical presentation and diagnosis of obstructive sleep apnea in adults. www.upto-date.com.

[bb0025] Young T., Palta M., Dempsey J., Peppard P.E., Nieto F.J., Hla K.M. (2009). Burden of sleep apnea: Rationale, design, and major
findings of the Wisconsin Sleep Cohort study. WMJ.

[bb0030] Peppard P.E., Young T., Barnet J.H., Palta M., Hagen E.W., Hla K.M. (2013). Increased prevalence of sleep-disordered breathing in adults. Am J Epidemiol.

[bb0035] Tufik S., Santos-Silva R., Taddei J.A., Bittencourt L.R. (2010). Obstructive sleep apnea syndrome in the Sao Paulo Epidemiologic Sleep Study. Sleep Med.

[bb0040] Bixler E.O., Vgontzas A.N., Lin H.M., Ten Have T., Rein J., VelaBueno A. (2001). Prevalence of sleep-disordered breathing
in women: Effects of gender. Am J Respir Crit Care Med.

[bb0045] Franklin K.A., Lindberg E. (2015). Obstructive sleep apnea is a common disorder in the population-a review on the epidemiology of sleep apnea. J Thorac Dis.

[bb0050] Jennum P., Riha R. (2009). Epidemiology of sleep apnoea/hypopnoea syndrome and sleep-disordered breathing. Eur Respir J.

[bb0055] Şahbaz S., İtil O., İnönü H., Öztura İ., Yemez B., Baklan B. (2008). Quality of life, frequency of anxiety and depression in
obstructive sleep apnea syndrome. Turkish Thoracic J.

[bb0060] Bayram N.A., Çiftçi B., Güven S.F., Bayram H., Diker E. (2007). Relationship between the severity of obstructive sleep apnea and hypertension. Anatolian J Cardiol.

[bb0065] Peppard P.E., Young T., Barnet J.H., Palta M., Hagen E.W., Hla K.M. (2013). Increased prevalence of sleep-disordered breathing
in adults. Am J Epidemiol.

[bb0070] Young T., Skatrud J., Peppard P.E. (2004). Risk factors for obstructive sleep apnea in adults. JAMA.

[bb0075] Epstein J., Kristo D., Strollo P.J. Jr., Friedman N., Malhotra A., Patil S.P. (2009). Adult obstructive sleep apnea task force of
the American Academy of Sleep Medicine. Clinical guideline for the evaluation, management and long-term care of
obstructive sleep apnea in adults. J Clin Sleep Med.

[bb0080] Hoffstein V., Mateika S. (1992). Differences in abdominal and neck
circumferences in patients with and without obstructive
sleep apnoea. Eur Respir J.

[bb0085] Katz I., Stradling J., Slutsky A.S., Zamel N., Hoffstein V. (1990). Do patients with obstructive sleep apnea have thick necks?. Am Rev Respir Dis.

[bb0090] Uyar M., Elbek O., Bayram N., Çiftçi N., Fakılı F., Aydın N. (2007). Risk Factors and Comorbid Diseases in Obstructive Sleep Apnea Syndrome. Turkish Thoracic Journal 10th
Annual National Congress; Antalya, Türkiye.

[bb0095] Duran J., Esnaola S., Rubio R., Iztueta A. (2001). Obstructive sleep
apnea-hypopnea and related clinical features in a population based sample of subjects aged 30 to 70 yr. Am J Respir Crit Care Med.

[bb0100] Meslier N., Lebrun T., Grillier-Lanoir V., Rolland N., Henderick C., Sailly J.C. (1998). A French survey of 3,225 patients treated
with CPAP for obstructive sleep apnea: Benefits, tolerance,
compliance, and quality of life. Eur Respir J.

[bb0105] Sawyer A.M., Gooneratne N.S., Marcus C.L., Ofer D., Richards K.C., Weaver T.E. (2011). A systematic review of CPAP adherence
across age groups: Clinical and empiric insights for developing CPAP adherence interventions. Sleep Med Rev.

[bb0110] Antic N.A., Catcheside P., Buchan C., Hensley M., Naughton M.T., Rowland S. (2011). The effect of CPAP in normalizing
daytime sleepiness, quality of life, and neurocognitive
function in patients with moderate to severe OSA. Sleep.

[bb0115] Bratton D.J., Stradling J.R., Barbé F., Kohler M. (2014). Effect of CPAP
on blood pressure in patients with minimally symptomatic
obstructive sleep apnoea: A meta-analysis using individual
patient data from four randomised controlled trials.. Thorax.

[bb0120] Campos-Rodriguez F., Martinez-Alonso M., Sanchez-de-la-Torre M., Barbe F. (2016). Spanish Sleep Network. Long-term
adherence to continuous positive airway pressure therapy
in non-sleepy sleep apnea patients. Sleep Med.

[bb0125] Shapiro G.K., Shapiro C.M. (2010). Factors that influence CPAP
adherence: An overview. Sleep Breath.

[bb0130] Budhiraja R., Parthasarathy S., Drake C.L., Roth T., Sharief I., Budhiraja P. (2007). Early CPAP use identifies subsequent
adherence to CPAP therapy. Sleep.

